# A Truncated Receptor TrkB Isoform (TrkB.T1) in Mechanisms of Genetically Determined Depressive-like Behavior of Mice

**DOI:** 10.3390/biomedicines11092573

**Published:** 2023-09-19

**Authors:** Marah Alsalloum, Tatiana Ilchibaeva, Anton Tsybko, Dmitry Eremin, Vladimir Naumenko

**Affiliations:** 1 Federal Research Center Institute of Cytology and Genetics, Siberian Branch of Russian Academy of Sciences, Prospekt Akad. Lavrentyeva 10, Novosibirsk 630090, Russia; alsallummm@bionet.nsc.ru (M.A.); eremin@bionet.nsc.ru (D.E.); naumenko2002@mail.ru (V.N.); 2 Department of Natural Sciences, Novosibirsk State University, Pirogova Street 2, Novosibirsk 630090, Russia

**Keywords:** truncated TrkB, TrkB.T1, BDNF, depressive-like behavior, aggressive behavior, AAV-mediated overexpression

## Abstract

Depression is a mental disorder that significantly reduces quality of life, and the discovery of new drug targets is an urgent problem for modern neuroscience. Brain-derived neurotrophic factor (BDNF) and its receptors have been found to participate in mechanisms of depression and antidepressant drugs’ action. In this study, we focused on a less-studied truncated isoform of receptor TrkB: TrkB.T1. Initially, we noticed that the level of TrkB.T1 is low in the hippocampus of Antidepressant-Sensitive Cataleptics (ASC) mice, which are characterized by genetically determined depressive-like behavior in contrast to “normal” C57BL/6J mice. Next, overexpression of TrkB.T1 receptor in hippocampal neurons of ACS mice was induced to clarify the role of this receptor in mechanisms of depressive-like behavior. TrkB.T1 overexpression lowered BDNF protein concentration in the hippocampus. On the behavioral level, TrkB.T1 overexpression severely decreased aggression and enhanced social behavior. Additionally, this excess of receptor TrkB.T1 slightly promoted anxiety and depressive-like behavioral traits without affecting learning and memory. Our results show that this TrkB isoform participates in the control of aggression, anxiety, and depressive-like behavior and in the regulation of BDNF system functioning in ASC mice (genetically predisposed to depressive-like behavior). Considering our findings, we believe that hippocampal receptor TrkB.T1 can be a drug target for the correction of behavioral pathologies.

## 1. Introduction

The Trk family of receptors includes TrkA, TrkB, and TrkC, which mediate the cellular effects of neurotrophins [1]. TrkB is a receptor for brain-derived neurotrophic factor (BDNF) and neurotrophin 4 (NT4), although it binds to neurotrophin 3 (NT3) too, albeit with weak affinity [2]. There are four major TrkB isoforms resulting from different splice variants of the *Ntrk2* transcript. These isoforms are the full-length tyrosine kinase receptor (TrkB.FL), the C-terminally truncated receptor (TrkB.T1), a C-terminally truncated Shc+ receptor (TrkB.shc), and a C-terminally truncated receptor retaining tyrosine kinase activity (TrkB-T-TK) [3].

Lacking the intracellular domain, TrkB.T1 cannot transduce signals to intracellular BDNF-initiated pathways. Nonetheless, TrkB.T1 has been found to have many functions. Having an extracellular domain identical to that of TrkB.FL, TrkB.T1 can bind BDNF with the same affinity and form homo/heterodimers with receptors TrkB.T1 and TrkB.FL [3]. TrkB.T1 heterodimers with TrkB.FL prevent cross-autophosphorylation between these two receptors. Thus, receptor TrkB.T1 acts as a dominant-negative inhibitor of receptor TrkB.FL and prevents the activation of PI3K–AKT, ERK–MAPK, and PLCγ pathways [4]. It is noteworthy that TrkB.T1 is also thought to be a dominant-negative inhibitor of p75^NTR^ [5], which is a well-known receptor for immature neurotrophin forms including proBDNF. Additionally, TrkB.T1 has a role in the regulation of BDNF levels in astrocytes by sequestration and translocation [3]. Receptor TrkB.T1 is reported to induce neurite outgrowth via a ligand-independent mechanism [6]. In the BDNF-dependent way, TrkB.T1 induces PKC and G protein activation, which causes taking the glial cell phenotypes by cortical neural stem cells [3]. In glial cells, receptor TrkB.T1 takes part in the actin cytoskeleton rearrangement and subsequent morphological changes mediated by its binding partner Rho GDP dissociation inhibitor (RhoGDI1) [7].

Since its discovery, studies on receptor TrkB.T1 have revealed its association with anxiety [8], aggression [9], social defeat stress [10], coordination of actions and habits [11], and long-term spatial memory [12]. Nevertheless, the role of TrkB.T1 in depressive-like behavior is yet to be evaluated. Furthermore, the modulation of receptor TrkB.T1 has been investigated in the whole brain in most of such studies, making it impossible to identify brain region–specific or cell-specific effects of TrkB.T1 overexpression/deletion. Considering that the hippocampus, especially its dorsal part, is a critical structure for spatial navigation [13], learning [14], memory [15], as well as regulation depressive-like behavior [16] and anxiety [17], it is of great interest to study functions of TrkB.T1 in the regulation of hippocampus-dependent behavior.

To discover the participation of receptor TrkB.T1 in depressive-like behavior, here we chose the Antidepressant-Sensitive Catalepsy (ASC) mouse strain: a genetic model of depression that has been developed by backcrossing predisposed-to-catalepsy CBA/Lac mice and catalepsy-prone AKR/J mice [18,19]. ASC mice show increased immobility in both the forced swim test (FST) and tail suspension test (TST) and decreased locomotor activity in the open field test as compared to parental strains [18]. They also show a weakened immune response [20,21] and a deficit of sexual motivation [22]. Administration of a recombinant BDNF protein into brain lateral ventricles of ASC mice reduces catalepsy and immobility time in the TST and raises the expression of genes encoding tryptophan hydroxylase 2 (Tph2) and 5-HT_2A_ receptor. On the other hand, BDNF receptors have not been studied in this model yet.

In this work, we assessed the effects of TrkB.T1 overexpression in hippocampal neurons on different kinds of behavior using a wide range of behavioral tests (open field test, elevated plus maze test, resident–intruder test, and Morris water maze, TST, and FST) as well as on the expression of key genes of BDNF systems (*Bdnf*, *Ntrk2*, and *Ngfr*) and on mRNA levels of *Mapk3*, *Creb1*, and early response genes (*Fos* and *Jun*) required for long-term potentiation (LTP) induction in the hippocampus of ASC mice, which show genetically determined depressive-like behavior.

## 2. Materials and Methods

### 2.1. Animals

**Figure 1:**
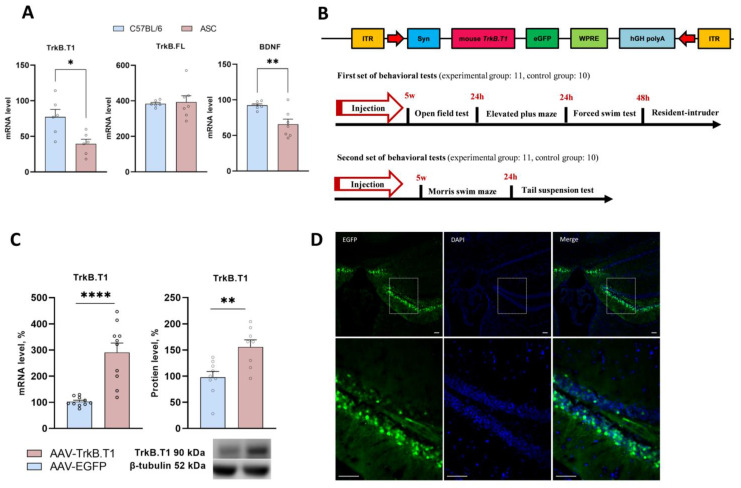
(**A**) Transcription of BDNF and its full and truncated receptors in ASC and C57BL/6 mice. (**B**) Schemes of behavioral test designs and AAV-TrkB.T1 plasmid design. (**C**) TrkB.T1 overexpression in the HC of ASC mice. Each mRNA level is presented as the ratio of the TrkB.T1 mRNA level to the rPol2 mRNA level. Protein level is presented as the ratio of the chemiluminescence intensities of the target protein to GAPDH. (**D**) Micrographs of brain slices after AAV-TrkB.T1-EGFP injection (scale bar = 100 µm). Data are presented as mean ± SEM (for AAV-EGFP, *n* = 11; for AAV-TrkB.T1, *n* = 10). * *p* < 0.05, ** *p* < 0.01; **** *p* < 0.0001.

Experiments were carried out on mature male mice of ASC strain (weight 25 ± 1 g). All experimental animals were 2 months old. Behavioral tests were divided into two series, and the first one included the open field test, elevated plus maze, FST, and resident–intruder test. The second series consisted of the Morris water maze test and TST. In each test series, two groups of mice were evaluated: an experimental group (11 mice) and a control group (10 mice). An outline of the experiment is shown in Figure 1B.

In the resident–intruder test, 1.5-month-old mice of the CD1 strain (weight 25 ± 1 g) were used aside from ASC mice. Mature male mice of C57BL/6J strain (2 months old; weight 25 ± 1 g) were used for comparative assessment of BDNF, TrkB, and TrkB.T1 mRNA levels. The animals were kept in the vivarium of the Institute of Cytology and Genetics SB RAS under standard laboratory conditions with a 12/12 h light/dark cycle and free access to feed and water.

For fluorescence imaging, we employed three mice injected with adeno-associated virus (AAV) vector AAV-TrkB.T1-EGFP.

### 2.2. Plasmids

With primers (forward, 5′-tagctGGATCCccaccATGTCGCCCTGGCTGAAGTG-3′; reverse, 5′-tagctACCGGTCTACCCATCCAGTGGGATCTTATGAAACAAAACAAA-3′) containing recognition sites of restriction endonucleases BamHI and AgeI, a gene corresponding to TrkB.T1 was synthesized by means of murine cDNA as a template. The obtained sequence was digested with restriction endonucleases BamHI and AgeI (New England Biolabs, Ipswich, MA, USA) and ligated into the pAAV_Syn_EGFP vector to be expressed under the control of a synapsin (Syn) promoter [23]. Sanger sequencing was used for verification of all cloning steps.

### 2.3. Cell Culture and Transfection

HEK293FT cells were subcultured in Dulbecco’s modified Eagle’s medium (DMEM) (Sigma-Aldrich, Darmstadt, Germany) supplemented with 10% of fetal bovine serum (Gibco, Waltham, MA, USA), 1% of GlutaMAX (Gibco, USA), and 1% of a penicillin–streptomycin solution (Gibco, USA). The cells were incubated at 37 °C with 5% CO_2_. The cells were split at 70% confluency, and the medium was refreshed every 2–3 days. HEK293FT cells were transfected with plasmids using polyethylenimine (PEI, Polysciences, 23966-2, Warrington, PA, USA), following the manufacturer’s instructions.

### 2.4. Production of rAAV Vectors

Packaging of plasmid AAV_Syn_TrkB.T1 or control plasmid pAAV_Syn_EGFP DNA into AAV capsids was performed by cotransfection of HEK-293FT cells with plasmids AAV-DJ (scAAV-DJ expression system by Cell Biolabs, Inc., San Diego, CA, USA) and pHelper (Cell Biolabs, Inc., USA) [23]. Harvesting of viral particles was performed in 48 h, in accordance with the protocol described by Grimm et al. [24]. The amount of the obtained viral particles was determined by real-time quantitative PCR (with primers 5′-cctggttgctgtctctttatgagg-3′ [forward] and 5′-tgacaggtggtggcaatgc-3′ [reverse]). A series of dilutions of an original plasmid of known concentration served as standards for determining the number of viral particles. Both AAV vectors utilized in this study had similar genomic titers (10^9^ viral genomes per microliter).

### 2.5. Stereotaxic Microinjections

Animals were anesthetized with a solution (1 mL/kg i.p.) of 2,2,2-tribromoethanol (Sigma-Aldrich, Germany) in 2-methyl-2-butanol (Sigma-Aldrich, Germany) and placed in a stereotaxic frame (TSE systems, Berlin, Germany). The skull was drilled bilaterally (AP: −2.0 mm, ML: −1.0 mm, DV: 1.5 mm and AP: −2.0 mm, ML: +1.0 mm, DV: 1.5 mm; http://labs.gaidi.ca/mouse-brain-atlas/?ml=-1.5&ap=-2&dv=2, accessed on 2 September 2021). Virus AAV_Syn_TrkB.T1 (AAV-TrkB.T1) or AAV_Syn_EGFP (control) was injected into the hippocampus bilaterally (1 μL per side) through a Hamilton syringe at a rate of 0.1 μL/min over a 10 min period. To minimize any drawback of the virus as the needle was removed, the needle was kept in place for further 2 min after the injection. After injections, the incision was closed with interrupted silk sutures, and the animal was placed in a warm cage.

### 2.6. Behavioral Testing

After 5 weeks of postoperative recovery, the mice underwent a behavioral test battery according to the experimental design shown in Figure 1B.

*Open field test*. The test setup represented a round arena 55 cm in diameter with walls 30 cm high. The illumination of the arena was 300 lux. The light source was placed under the arena (inverted lighting), thereby improving the contrast of an animal against the background. Mice were placed near the wall of the arena and allowed to freely explore the space for 5 min. Using EthoStudio software and hardware, the following parameters were automatically estimated: total distance traveled (m) and time spent at the center of the arena (%), the diameter of which was 27.5 cm. The numbers of rearings and groomings as well as average grooming duration (s) were determined manually. After each mouse, the arena was wiped with wet and dry wipes.

*Elevated plus maze test*. The test setup consisted of four arms 30 cm long and 6 cm wide connected at a right angle and located at a height of 60 cm above the floor. One pair of arms had walls 20 cm high (closed arms), and the other pair had no walls (open arms). The arena was illuminated from above with diffused light. Mice were placed at the intersection of two arms, and movement was automatically recorded for 5 min using the EthoStudio software. The following parameters were automatically estimated: distance traveled (m), time spent in open and closed arms (%), and studied area of each arm (%). Peeks into closed arms and head dips were counted manually.

*The intermale aggression test*. The test was performed within the resident–intruder paradigm as described previously [25,26]. A random-bred adult male mouse of the albino CD1 strain (intruder) was introduced into a home cage of a tested male (resident). Each intruder was used no more than five times. The duration of the test was 10 min. A resident that did not attack the intruder during this time was considered nonaggressive. As soon as a fight began, the number and the duration of attacks were registered for 2 min in the EthoStudio software by an observer blinded to the animal’s treatment, after which the experiment was stopped. The number and duration of social interactions (e.g., biting and sniffing) were also recorded.

*Tail suspension test*. Mice were fixed with adhesive tape by the tail and hung on a horizontal bar at a height of 30 cm from a table surface. The number of immobilization episodes and latency time and duration of each episode were recorded using the EthoStudio software.

*Forced swim test*. Mice were placed in a clear plastic box (15 × 25 cm) filled with water at 25 °C. After 2 min of adaptation, total immobility time and latency time of the first episode of immobility were recorded in the EthoStudio software Version 2.0 during 4 min [27].

*Morris water maze test*. The setup comprised a round pool 110 cm in diameter with walls 30 cm high. The pool was filled with water (temperature 25 °C, water column height ~15 cm), and powdered milk was added to make it opaque. We tracked each animal by transmitted (inverted)-lighting techniques developed for the open field test. In the EthoStudio software, the pool was divided into four sectors. A glass round escape platform (5 cm diameter and 14.5 cm height) was located 1 cm below the water surface near the center of the lower right quadrant of the maze. A black rectangle measuring 6 × 12 cm (visual mark) was glued to the pool wall closest to the platform. Mice were automatically tracked by means of the EthoStudio software.

Each training period lasted 4 days, and each session contained three trials. During the training, mice were allowed to search for the platform for 60 s. At the end of each test, mice were placed on the platform and held (if necessary) there for 15 s. Measured parameters were latency to find the platform (s), distance traveled (m), and total distance to the platform (m), which was calculated as the sum of distances from the mouse to the platform at each time point. The average value of three tests was subjected to further analyses.

On the fifth day, the platform was removed, mice were placed at the center of the pool, and their movements were recorded for 60 s. Time spent in the target sector (%) was estimated, and then the average value for the three trials was calculated. A statistically significant excess of time spent in the target sector over a random level (25%) indicated that the mouse remembered the location of the platform.

### 2.7. Excision of the Brain Structures

Two days after the behavioral testing, the animals were decapitated, and hippocampi were isolated on ice, frozen in liquid nitrogen, and stored at −80 °C until subsequent procedures.

### 2.8. Reverse–Transcription Quantitative PCR

We mixed a 1 µL aliquot of cDNA with 2 µL of PCR buffer (containing SYBR green I), 2 µL of 25 mM dNTP, 2 µL of 25 mM MgCl_2_, 2 µL of mixture of forward and reverse primers, and 0.16 µL of Taq DNA polymerase, and adjusted the volume to 20 µL with deionized H_2_O. Two types of standard were used for quantitative analysis of mRNA levels: external and internal. An internal standard (mRNA of the *rPol2* gene) was employed to make sure that the reverse transcription worked and as a basis for calculating mRNA level of the studied genes. In preliminary experiments, no differences were found in the level of *rPol2* mRNA among the control and experimental groups. A series of dilutions of DNA (of a known concentration) isolated from the mouse liver served as an external standard, which made it possible to check whether the PCR worked and to determine the number of mRNA copies of the studied genes in the samples. Gene expression is presented as the ratio of cDNA of an analyzed gene to 100 copies of *rPol2* cDNA [28]. Melting curve analysis was performed after each run to control PCR specificity. Primers used for PCR and their sequences and annealing temperatures are shown in Table 1; they were selected based on the sequences published in the EMBL Nucleotide database and were synthesized in the Laboratory of Synthetic Biology at the Institute of Chemical Biology and Fundamental Medicine SB RAS (Novosibirsk).

### 2.9. Western Blot

Membrane proteins were isolated by homogenizing samples in a buffer consisting of 300 mM sucrose, 10 mM Tris-HCl pH 7.2, 1 mM EDTA, 5 mM β-mercaptoethanol, and protease inhibitors (GE Healthcare, Chicago, IL, USA). Then, the samples were centrifuged at 500× *g* for 15 min at 4 °C, the supernatant was collected and centrifuged at 20,000× *g* at 4 °C for an hour. The cytosolic fraction was collected, and the precipitate remaining in the tube was resuspended in homogenizing buffer (see above). Both fractions were stored at −80 °C. Protein concentration was assessed spectrophotometrically using the Pierce BCA Protein Assay Kit (Thermo Fisher Scientific Inc., Waltham, MA, USA) and a NanoDrop 2000C spectrophotometer (Thermo Fisher Scientific Inc., USA). The samples were adjusted to equal concentrations (1 mg/mL) using 2× Laemmli’s buffer and heat denatured for 10 min at 95 °C. Extracts (30 μg of total protein per lane for BDNF analysis and 15 μg of total protein per lane for analysis of other proteins) separated by SDS-PAGE were blotted onto a nitrocellulose membrane (Bio-Rad Laboratories, Inc., Hercules, CA, USA) with the help of a Trans-Blot Turbo Transfer System (Bio-Rad Laboratories, Inc., USA). The membranes were blocked in Tris-buffered saline supplemented with 0.05% Tween 20 (TBST) containing 5% of nonfat dry milk (NFDM-TBST) for 1 h, washed, and then incubated with primary antibodies shown in Table 2. After protein detection, all blots were stripped and then re-probed with a horseradish peroxidase-conjugated anti-GAPDH antibody as a loading control. To detect a protein, the membranes were washed with TBST (5 × 5 min) followed by incubation with a secondary antibody conjugated with horseradish peroxidase. After washing, the blots were treated with the Clarity Western ECL substrate (Bio-Rad Laboratories, Inc., USA). Protein bands were detected on a C-DiGit Blot Scanner (LI-COR, Lincoln, NE, USA) and were quantified by volumetric densitometry in ImageStudio software v.5.5.4 (LI-COR Image Studio Software, Lincoln, NE, USA). Protein expression is expressed in relative units normalized to the expression of GAPDH or β-tubulin.

### 2.10. Fluorescence Microscopy of Mouse Brain Sections

In the fifth week after injection, mice were transcardially perfused with phosphate-buffered saline (PBS) and a 4% paraformaldehyde solution. Brains were excised, postfixed with 4% paraformaldehyde for 6 h, and immersed in 20% sucrose in PBS for 2 days. Using a cryostat (Thermo Fisher Scientific, Inc., Waltham, MA, USA), 14 µm slices were obtained, and finally mounted in an antiquenching medium with DAPI (ab104139; Abcam, UK) followed by examination under a Zeiss AxioImager2 microscope with 10× and 40× air-immersion objectives. The mice used for the imaging were not subjected to the behavioral testing.

### 2.11. Statistics

All data were tested for normality of distribution by Shapiro–Wilk, Kolmogorov–Smirnov, D’Agostino and Pearson, and Anderson–Darling tests. Gene expression data and the results of open field, elevated plus maze, tail suspension, forced swim, and resident–intruder tests were processed by Student’s *t* test if the data were normally distributed. Nonparametric Mann–Whitney U test was utilized when data were non-normally distributed. The results of the Morris water maze test were subjected to repeated measures analysis of variance (ANOVA) with Fisher’s test post hoc comparison. Analysis of covariance (ANCOVA) was applied to determine whether immobility in the tail suspension test and social activity in the resident–intruder test depended on reduced locomotor activity in the open field test.

## 3. Results

### 3.1. The mRNA Level of Receptor TrkB.T1 in the Hippocampus of ASC Mice Is Lower as Compared to “Normal” Mice

In a preliminary series of experiments, we compared mRNA levels for BDNF and its receptors (TrkB.FL and TrkB.T1) in the hippocampus of “depressive” (ASC) and “nondepressive” (C57BL/6J) mice. We revealed that the BDNF mRNA level (t(12) = 3.54, *p* = 0.004) was reduced in the hippocampus of ASC mice, in agreement with reports on reduced BDNF expression in patients with major depressive disorder [29]. Of note, while the TrkB.FL mRNA level remained the same in both mouse strains (t(11) = 0.24, *p* = 0.81), the TrkB.T1 mRNA level proved to be downregulated (t(10) = 3.08, *p* = 0.01) in the hippocampus of ASC mice compared to C57BL/6J mice (Figure 1A). As far as we know, this is the first animal model of depression indicating the disrupted expression of a truncated isoform of receptor TrkB.

To identify the role of hippocampal TrkB.T1 in mechanisms underlying depressive-like behavior of ASC mice, we induced TrkB.T1 overexpression by injecting the hippocampi with a recombinant AAV carrying the gene expressing TrkB.T1. Five weeks after the injection, we performed behavioral testing, and next confirmed TrkB.T1 overexpression in the hippocampus both at mRNA (t(18) = 5.22, *p* < 0.0001) and protein levels (t(15) = 3.37, *p* = 0.004; Figure 1C).

### 3.2. TrkB.T1 Overexpression in the Hippocampus of ASC Mice Lowers Locomotor Activity and Has Weak Effects on Anxiety and Exploratory Activity

Mice with TrkB.T1 overexpression spent significantly less time at the center of the arena in the open field test (t(19) = 2.48, *p* = 0.02) (Table 3). This finding suggests that TrkB.T1 overexpression may heighten anxiety in “depressive” animals. Nonetheless, this result was not confirmed by the elevated plus maze test, where the two groups of mice spent similar time in closed arms (U = 50, *p* = 0.75); the same was true for the time spent in open arms (t(18) = 1.25, *p* = 0.40).

Mice with TrkB.T1 overexpression also demonstrated fewer rearings in the open field test (t(17) = 3.46, *p* = 0.003). On the other hand, the elevated plus maze did not reveal any differences in exploratory activity, assessed by means of peeks (t(18)= 0.83, *p* = 0.41), latency to the first peek (U = 40, *p* = 0.46) and the number of head dips (U = 54.5, *p* = 0.98) (Table 4).

Mice with TrkB.T1 overexpression also showed a strong tendency toward shorter distance traveled in the open field test (t(19) = 2.00, *p* = 0.05). This result was confirmed by the elevated plus maze test (t(18) = 2.26, *p* = 0.036).

Other parameters analyzed in both tests were not affected by TrkB.T1 overexpression: explored area in the open field test (U = 39, *p* = 0.28), explored area in closed arms of the elevated plus maze (U = 30, *p* = 0.08), explored area in open arms (U = 42, *p* = 0.54), and grooming parameters (the count (U = 43, *p* = 0.92), duration (U = 38, *p* = 0.40), and latency time of the first grooming episode (t(19) = 0.16, *p* = 0.16)).

### 3.3. TrkB.T1 Overexpression in the Hippocampus of ASC Mice Has a Weak Prodepressive Effect

Mice with TrkB.T1 overexpression showed longer immobility time as compared to the control (t(21) = 2.61, *p* = 0.01) in the tail suspension test (Table 5). Nevertheless, latency to the first immobility episode (U = 57, *p* = 0.59) and the number of immobility episodes (t(21) = 0.51, *p* = 0.61) did not differ between the two groups. To investigate whether immobility in the tail suspension test depends on diminished locomotor activity, ANCOVA was performed with total traveled distance in the open field test being a continuous predictor of immobility in the tail suspension test. In this case, AAV-TrkB.T1-EGFP–injected mice still demonstrated a significantly higher level of immobility in the tail suspension test as compared to corresponding control animals (F1,18 = 5.30, *p* = 0.03).

Because the influence of receptor TrkB.T1 on immobility was not confirmed in the forced swim test (immobility duration (t(14) = 0.54, *p* = 0.70), latency to the first immobility episode (t(16) = 1.73, *p* = 0.10), and the number of immobility episodes (t(16) = 0.34, *p* = 0.10)), we assumed that the TrkB.T1-induced prodepressive effect is too weak to be detected by both tests.

### 3.4. TrkB.T1 Overexpression in the Hippocampus of ASC Mice Decreases Aggression and Improves Social Behavior

**Figure 2:**
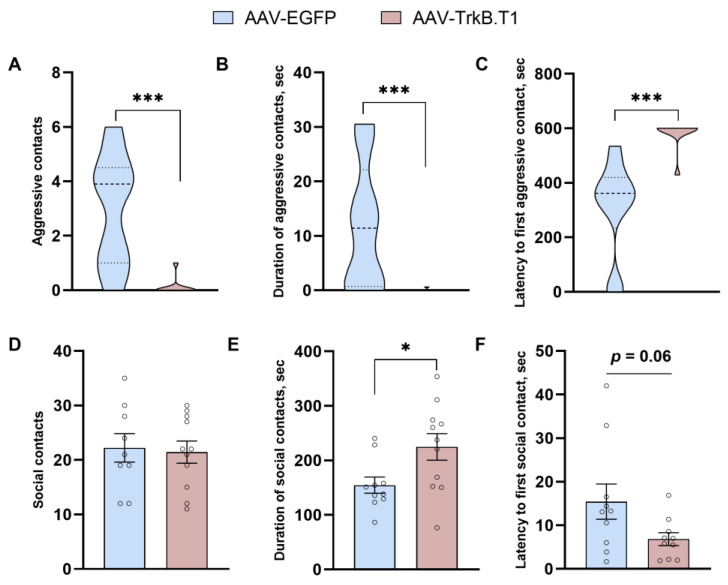
Effects of TrkB.T1 overexpression in the hippocampus of ASC mice on aggressiveness and social behavior assessed by the resident–intruder test. * *p* < 0.05, *** *p* < 0.001 vs the AAV-EGFP group. Data are presented as violin plot for (**A**–**C**) and mean ± SEM for (**D**–**F**) (for AAV-EGFP, *n* = 10; for AAV-TrkB.T1, *n* = 10).

The resident–intruder test revealed that TrkB.T1 overexpression significantly diminishes the number of aggressive contacts (U = 6.5, *p* = 0.0004; Figure 2A) and duration of aggressive contacts (U = 6.5, *p* = 0.0003; Figure 2B), and extends the latency time of the first aggressive contact (U = 2, *p* < 0.0001; Figure 2C). This effect on aggression was accompanied by a prosocial effect reflected in significantly prolonged duration of social contacts of mice in the experimental group compared with the control (t(18) = 3.26, *p* = 0.004). The number of social contacts (t(18) = 0.23, *p* = 0.63) was not significantly affected by TrkB.T1 overexpression. At the same time, latency to the first social contact (t(18) = 2.00, *p* = 0.06) manifested a strong decreasing trend in the experimental group. In ANCOVA with traveled distance as a continuous predictor, we also detected a significant impact of TrkB.T1 overexpression on the number (F1,16 = 18.45, *p* = 0.0004), duration (F1,17 = 7.08, *p* = 0.016), and latency time (F1,18 = 10.51, *p* = 0.004) of aggressive contacts and on the duration of social interactions (F1,17 = 7.41, *p* = 0.014).

### 3.5. TrkB.T1 Overexpression in the Hippocampus of ASC Mice Has No Influence on Learning and Spatial Memory

**Figure 3:**
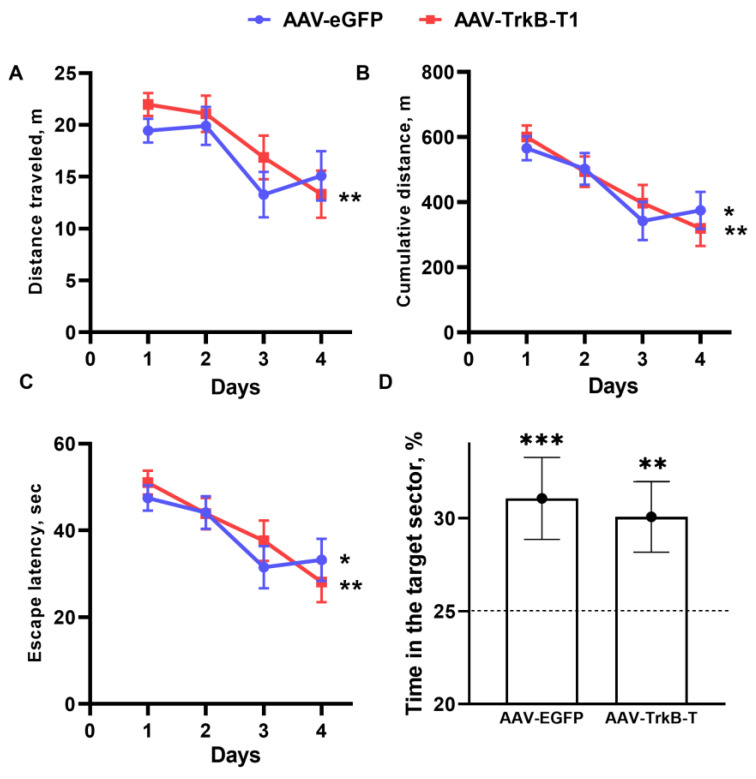
Spatial learning in the Morris water maze in ASC mice featuring TrkB.T1 overexpression. Dynamics of the distance traveled (**A**), distance between the mouse and the platform throughout each trial (**B**), and escape latency (**C**) during the acquisition period. (*n* = 10) * *p* < 0.05; ** *p* < 0.01; *** *p* < 0.001 vs. the first day of the acquisition period. (**D**) Time spent in the target sector on the fifth day. The values are the means of corresponding three trial results, ** *p* < 0.01, *** *p* < 0.001 vs. a random level (25%) marked with dotted line.

We carried out the Morris water maze test to evaluate the effects of receptor TrkB.T1 on learning and memory (Figure 3). All parameters examined throughout the learning period declined significantly with time (latency to find the platform (*p* < 0.05 and *p* < 0.001 for groups AAV-EGFP and AAV-TrkB.T1), distance traveled by a mouse (*p* < 0.01 and *p* < 0.05 for groups AAV-EGFP and AAV-TrkB.T1), and cumulative distance between the mouse and the platform at each time point (*p* < 0.05 and *p* < 0.01 for groups AAV-EGFP and AAV-TrkB.T1)), indicating that both groups successfully learned the task. For each examined parameter, we found a significant effect of the training day on the distance traveled (F3,63 = 7.19; *p* < 0.001), cumulative distance (F3,63 = 10.48; *p* < 0.001), and latency to find the platform (F3,63 = 8.94; *p* < 0.001).

By contrast, the influence of TrkB.T1 overexpression was not significant (distance traveled (F1,21 = 0.83; *p* ˃ 0.05), cumulative distance (F1,21 = 0.02; *p* ˃ 0.05), and latency to find the platform (F1,21 = 0.13; *p* ˃ 0.05)). This meant that the two groups had a similar ability to learn, and the same was true for the ability to memorize.

On retention day (the test without the platform), both groups spent significantly more time in the target sector compared to the other sectors (AAV-EGFP (*p* < 0.001) and AAV-TrkB.T1 (*p* < 0.01)) and the influence of training was confirmed (F1,42 = 23.64; *p* < 0.001). Therefore, the effect of TrkB.T1 overexpression on spatial memory and learning was not detectable (F1,42 = 0.05; *p* ˃ 0.05).

### 3.6. Overexpression of Receptor TrkB.T1 Further Lowers the BDNF Level in the Hippocampus of ASC Mice but Does Not Affect the Transcription of Genes Involved in the Induction of LTP

**Figure 4:**
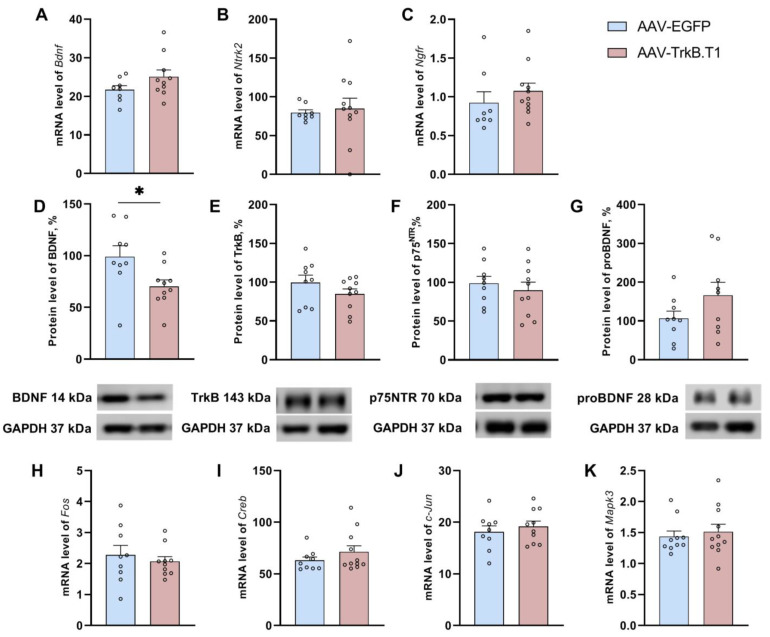
Effects of TrkB.T1 upregulation on the expression of the key genes of the BDNF system (**A**–**G**) and on the expression of genes involved in the induction of LTP (**H**–**K**). The mRNA levels are presented as the ratio of target mRNA level of to the *rPol2* mRNA level. Protein levels are presented as the ratio of chemiluminescence intensity of a protein to that of GAPDH. * *p* < 0.05 vs the AAV-EGFP group. Data are presented as mean ± SEM (n_AAV-EGFP_ = 10, n_AAV-TrkB.T1_ = 10).

TrkB.T1 overexpression significantly lowered the BDNF protein level in mice of the experimental group (t(17) = 2.39, *p* = 0.02), while transcription of its gene remained unchanged (t(16) = 1.56, *p* = 0.13; Figure 4). This finding possibly indicated a role of TrkB.T1 in the regulation of BDNF at the protein level. Expression of receptor TrkB-FL encoded by the *Ntrk2* gene (mRNA (t(15) = 0.48, *p* = 0.63) and protein (t(17) = 1.29, *p* = 0.21)) was not affected by TrkB.T1 overexpression. Neither was the expression of the receptor of proBDNF, i.e., receptor p75^NTR^ encoded by the *Ngfr* gene (mRNA (U = 25, *p* = 0.12), protein (t(17) = 0.64, *p* = 0.52); Figure 4C,F).

We did not find any impact of TrkB.T1 overexpression on mRNA levels of LTP-related genes: Creb1 (t(18) = 1.15, *p* = 0.26), Mapk3 (U = 47, *p* = 0.60), and early response genes: Fos (t(17) = 0.61, *p* = 0.54) and Jun (t(17) = 0.67, *p* = 0.50; Figure 4).

## 4. Discussion

Our preliminary study suggests that transcription of genes of TrkB.T1 and BDNF is significantly lower in the hippocampus of ASC mice (which are genetically predisposed to depressive-like behavior) than in “nondepressive” C57BL/6J mice. Intrigued by this novel finding, we induced hippocampal TrkB.T1 overexpression in ASC mice using an AAV-TrkB.T1 construct. Then, we analyzed behavioral patterns and assessed the expression of genes that may be responsible for changes in behavior. Overexpression of TrkB.T1 did not cause regulatory compensation in terms of receptor TrkB.FL expression in the hippocampus of the studied mice, implying that the observed behavioral and genetic alterations are TrkB.T1-induced.

Open field test results showed that overexpression of TrkB.T1 receptor has a mild effect on anxiety, judging by less time spent at the center of the arena and more time spent near the wall of the arena by mice from experimental group (TrkB.T1 overexpression). To rule out a possible false positive result, we measured the explored area of the open field in both groups. Because there were no differences in this parameter between the groups, it is safe to assume that the difference in time spent at the center is actually due to a difference in anxiety rather than changes in locomotor or exploratory activity. This result, however, failed to be replicated in the elevated plus maze test: the two groups spent similar periods in closed arms, and the same was true for open arms. Head dips, which are an indicator of anxiety [30], also did not differ between the groups in the elevated plus maze. The discrepancy between the open field and elevated plus maze tests is explained by the fact that each of them measures different, albeit partially overlapping, characteristics of anxiety [31].

We noticed the decrease in the number of rearings in TrkB.T1 overexpressed mice. Given that the explored area of arena was not changed, the decrease in the number of rearings in the open field test may also be due to greater anxiety [30]. In support of this notion, it was demonstrated recently that exploratory rearings are stress sensitive [32]. In addition, this is in a good concordance with decreased time spent at the center of the arena in the experimental group.

Of note, TrkB.T1 knockout mice tend to spend less time in the center of the arena in the open field test and spend significantly less time in the open arms of the elevated plus maze [8]. Thus, TrkB.T1 overexpression in our study and the TrkB.T1 knockout in the work of Carim-Todd et al. had partially similar effects on anxiety. Some discrepancies may be due to the TrkB.T1 knockout implemented by Carim-Todd and coauthors is not limited to the hippocampus alone.

In addition, the overexpression of TrkB.T1 reduced the distance traveled by the experimental group in the elevated plus maze. Mice with TrkB.T1 overexpression also tended to have a shorter distance traveled in the open field test. On the other hand, the locomotor activity measured by the Morris water maze test did not differ between the groups. It is noteworthy that the knockout of receptor TrkB.T1 in C57BL/6J mice does not affect their locomotor activity [8]. TrkB.T1 overexpression in another model does not affect locomotion either, as tested via fear conditioning [33]. While some articles suggest that changes in motor activity may reflect depressiveness and can be an indicator of the efficacy of antidepressants [34,35,36], we did not find any changes in explored area of arena both in open field and elevated plus maze tests, which not allow us to assume a decrease in locomotor activity and its link with depressive-like traits in experimental animals.

FST and TST are designed to assess depressive-like behavior in rodents. Our results uncovered an increase in immobility time in the TST but not in the FST during hippocampal TrkB.T1 overexpression. The discrepancy between these two closely related tests is explained by the fact that both tests, although similar in the constructs they are designed to assess, are probably different in terms of the biological substrates underlying the observed behavior [37]. Taking into account the fact that the duration of immobility is the main parameter measured in both tests, and that these tests have different sensitivity to the antidepressant effect that reduces immobility [37], it is likely that receptor TrkB.T1 has a prodepressive effect, but it is not strong enough to be detected by both tests.

The Morris water maze test revealed that hippocampal receptor TrkB.T1 does not affect learning dynamics and spatial memory in ASC mice. These results are consistent with unchanged mRNA levels of the *Mapk3*, *Creb1*, and early response genes (*Fos* and *Jun*) required for LTP induction [38,39]. Our results showing normal learning and memory functions after TrkB.T1 overexpression, along with normal expression of LTP-inducing genes, are consistent with the findings of Carim-Todd et al. and Saarelainen et al., who have demonstrated that TrkB.T1-overexpressing mice and TrkB.T1 knockout mice have normal LTP and do not experience memory or learning deficits tested in the Morris water maze [8,12]. On the contrary, the deletion of receptor TrkB.T1 affects LTP in mouse models of amyotrophic lateral sclerosis (ALS) [40]. In the context of mice with ALS, researchers propose that the TrkB.T1 deletion acts as an “LTP desaturation” strategy because receptor TrkB.T1 does not alter LTP under physiological conditions [40].

We found that the baseline level of BDNF in ASC mice is lower than that in “nondepressive” C57BL/6J mice. This is expected because a depressive phenotype is known to be associated with BDNF deficiency [29]. For example, patients with major depressive disorder have lower serum BDNF levels than do healthy controls [41]. Furthermore, in postmortem samples of the hippocampus from suicide victims, underexpression of BDNF and of its receptor TrkB has been reported repeatedly [42,43,44]. Notably, central administration of a recombinant BDNF protein causes a prolonged improvement of depression-like behavior in ASC mice [45]. In the current study, we showed that hippocampal overexpression of TrkB.T1 further reduced the BDNF level, in line with the increase in depressive-like behavioral traits observed in the tail suspension test and increase in anxiety. On the basis of these findings, we believe that the low TrkB.T1 level is an adaptation to an initially low level of BDNF seen in this model of depression. It is noteworthy that the observed change in the BDNF level was detectable only at the protein level (not at the mRNA level). The level of proBDNF was not affected either. These data indicate that TrkB.T1 regulates the presence of BDNF in the cytoplasm without interfering with its de novo synthesis and without regulating its precursor proBDNF. We can hypothesize that the underlying mechanism involves BDNF sequestration by receptor TrkB.T1. This function has been found only in non-neuronal cells [3]. Our results suggest that TrkB.T1 performs the same function in neuronal cells because the genetic construct used by us induced TrkB.T1 overexpression only in neuronal cells, owing to the synapsin promoter.

Additionally, we noticed a dramatic effect of receptor TrkB.T1 on aggression and social behavior. Overexpression of TrkB.T1 (i) reduced the number and duration of aggressive contacts, (ii) increased latency time of the first aggressive contact, (iii) extended the duration of social contacts, and (iv) diminished latency time of the first social contact. The antiaggressive effect of TrkB.T1 overexpression along with the decline of BDNF levels is consistent with the findings of Ilchibaeva and coauthors [9], who have demonstrated a decrease in TrkB.T1 levels and a high level of BDNF in the hippocampus of highly aggressive rats. Moreover, as previously reported, deletion of receptor TrkB.T1 in a heterozygous BDNF background partially rescues the enhanced aggression exhibited by BDNF heterozygous mice [8]. At the same time, some studies indicate associations of increased aggressiveness with elevation in truncated TrkB. In the study of Mikics and coauthors [46], aggressiveness in rats provoked by social isolation was accompanied by increase in TrkB.T1 level in the infralimbic cortex. Similarly, we previously demonstrated extremely high level of TrkB.T1 in the frontal cortex of highly aggressive rats [9]. Recently it was shown that expression of truncated TrkB restricted to parvalbumin interneurons in the medial prefrontal cortex resulted in neuronal disinhibition and increase in intermale aggression [47]. We propose that similarly to BDNF, the amount of receptor TrkB.T1 must be maintained at a certain level: an excess and deficiency (or functioning) can result in aggression [48,49]. This may largely depend on the brain structure and its local neural circuits. In addition, it is possible that the participation of TrkB.T1 in the regulation of aggression is not limited to simple downregulation of the TrkB signaling pathway or BDNF sequestration and may recruit other molecular mechanisms.

## 5. Conclusions

We showed that receptor TrkB.T1 is involved in mechanisms underlying anxiety, depressive-like behavioral traits, and aggression in ASC mice, which are genetically predisposed to depressive-like behavior. We confirmed for the first time that receptor TrkB.T1 regulates the BDNF level in neuronal cells. This knowledge means that TrkB.T1 should be taken into consideration in further studies on behavioral pathologies and underlying mechanisms.

## Figures and Tables

**Table 1:** The primer sequences, annealing temperatures, and PCR product lengths.

Target Gene	Primer Sequences	Annealing Temperature, °C	Amplicon Length, bp
*rPol2*	F 5′-TGTGACAACTCCATACAATGC-3′ R 5′-CTCTCTTAGTGAATTTGCGTACT-3′	60	188
*Bdnf*	F 5′-TGAAGCCACCTCTCTCAGTC-3′ R 5′-AGCAGTACTCTCAGGGCTAGG-3′	59	126
*Ngfr*	F 5′-CTTGCTTCTGACCACCCTTCTC-3′ R 5′-GCTCCCTTCTCCTAGTGTCAAAC-3′	61	145
*Ntrk2*	F 5′-GTCGCTTTCTCTCCCTTTGG-3′ R 5′-GCATTGTTAGTTGTGGTGGGC-3′	60	277
*TrkB.T1* cDNA	F 5′-ACCCAAATTACCCTGAAGTC-3′ R 5′-CCAGTGGGATCTTATGAAAC-3′	59	258
*Creb*	F 5′-GCTGGCTAACAATGGTACGGAT-3′ R 5′-TGGTTGCTGGGCACTAGAAT-3′	64	140
*Fos*	F 5′-AAAGAGAAGGAAAAACTGGAG-3′ R 5′-CGGAAACAAGAAGTCATCAA-3′	58	246
*Mapk3*	F 5′-TGCCCTCGAAAACCAAGGTG-3′ R 5′-CGAAGGTGAATGGCTCCTCG-3′	60	195
* c-Jun *	F 5′-CCCTGGCTGAACTGCATAGC-3′ R 5′-GTTGAAGTTGCTGAGGTTGGCC-3′	61	182

**Table 2:** Characteristics of antibodies used.

Antibodies	Dilution	Incubation	Diluent	Producer
*Primary antibodies*				
Rabbit polyclonal anti-BDNF antibody (EPR1292)	1:1000	Overnight, 4 °C	3% BSA in TBST	Abcam, Cambridge, UK (ab108319)
Mouse monoclonal anti-proBDNF antibody	1:2500	Overnight, 4 °C	5% NFDM-TBST	Santa Cruz, Santa Cruz, CA, USA (Sc 65513)
Rabbit polyclonal anti-TrkB antibody	1:400	Overnight, 4 °C	5% NFDM-TBST	Alomone, Jerusalem, Israel (#ANT-019)
Goat polyclonal anti-TrkB antibody	1:500	Overnight, 4 °C	3% BSA in TBST	R&D Systems, Minneapolis, MN, USA
Rabbit polyclonal anti-p75^NTR^ antibody	1:500	2 h, RT	5% NFDM-TBST	Abcam, UK (ab38335)
Mouse polyclonal anti-GAPDH antibody	1:10,000	Overnight, 4 °C	5% NFDM-TBST	Abcam, UK (Ab8245)
Goat polyclonal anti-tubulin antibody	1:20,000	2 h, RT	5% NFDM-TBST	Abcam, UK (ab69485)
*Secondary antibodies*				
Goat anti-rabbit IgG	1:10,000	1 h, RT	5% NFDM-TBST	Invitrogen, Waltham, MA, USA (G-21234)
Rabbit anti-mouse IgG	1:30,000	1 h, RT	3% BSA in TBST	Invitrogen, USA (#31430)
Donkey anti-goat IgG	1:5000	1 h, RT	5% NFDM-TBST	Santa Cruz, USA (Sc 2020)

**Table 3:** Characteristics of the behavior of ASC mice in the open field test after TrkB.T1 overexpression.

Characteristic	AAV-EGFP Group	AAV-TrkB.T1 Group
Time at the center of arena, %	17.49 ± 1.33	13.85 ± 0.69 *
Explored area of the arena, %	96.86 ± 0.72	96.45 ± 0.48
Distance traveled, m	29.00 ± 0.64	27.14 ± 0.66
Rearings, number	14.22 ± 0.84	8.90 ± 1.24 **
Number of grooming episodes	1.556 ± 0.24	1.60 ± 0.22
Duration of groomings, s	3.52 ± 0.80	8.57 ± 2.64
Latency to first grooming, s	112.0 ± 21.18	115.9 ± 12.72

* *p* < 0.05; ** *p* < 0.01 vs. the AAV-EGFP group. Data are presented as mean ± SEM (for AAV-EGFP, *n* = 10; for AAV-TrkB.T1, *n* = 11).

**Table 4:** Characteristics of the behavior of ASC mice in the elevated plus maze after TrkB.T1 overexpression.

Characteristic	AAV-EGFP Group	AAV-TrkB.T1 Group
Time spent in closed arms, %	83.83 ± 3.05	84.29 ± 4.14
Area explored in closed arms, %	77.24 ± 4.72	85.39 ± 0.73
Time spent in open arms, %	10.32 ± 2.27	6.75 ± 1.70
Area explored in open arms, %	63.41 ± 10.09	52.55 ± 9.07
Peeks count from closed arms	9.22 ± 0.46	8.36 ± 0.84
Latency to first peek from closed arms, s	16.07 ± 1.66	20.14 ± 3.07
Peeks count from arena edge	6.90 ± 2.27	3.90 ± 0.65
Distance traveled, m	5.95 ± 0.34	5.08 ± 0.19 *

* *p* < 0.05 vs. the AAV-EGFP group (for AAV-EGFP, *n* = 10; for AAV-TrkB.T1, *n* = 10).

**Table 5:** Characteristics of the behavior of ASC mice in the tail suspension test and forced swim test after TrkB.T1 overexpression.

Characteristic	AAV-EGFP Group	AAV-TrkB.T1 Group
*Tail suspension test*		
Immobility duration, s	99.22 ± 4.69	121.0 ± 6.69 *
Immobility episodes	8.818 ± 0.60	9.250 ± 0.57
Latency to first episode	138.3 ± 5.74	132.3 ± 3.06
*Forced swim test*		
Immobility duration, s	25.78 ± 5.10	21.86 ± 4.89
Immobility episodes	7.400 ± 0.76	7.000 ± 0.86
Latency to first episode	147.4 ± 5.71	183.9 ± 22.51

* *p* < 0.05 vs. the AAV-EGFP group. Data are presented as mean ± SEM (for AAV-EGFP, *n* = 10; for AAV-TrkB.T1, *n* = 11).

## Data Availability

Raw data are available from the corresponding author upon reasonable request.
